# 

**Published:** 1875-04

**Authors:** 


FOUR DOLLARS A YEAR, POSTAGE FREE. SINGLE COPIES, 35 CENTS.

VOL. XXXII. April, 1875. NUMBER 4.

THE

### Chicago Medical Journal.

J. ADAMS ALLEN, M.D., LL.D., WALTER HAY, M.D.,

EDITORS.

TWELVE TIMES A YEAR.

CHICAGO:

W. B. KEEN, COOKE & CO., PUBLISHERS,

NOS. 113 AND 115 STATE STREET.

Copyright, A.D. 1875. W. B. KEEN, COOKE & CO.

